# Dissolution of Al-Substituted Goethite in the Presence of Ferrichrome and Enterobactin at pH 6.5

**DOI:** 10.1007/s10498-016-9304-4

**Published:** 2016-10-12

**Authors:** William E. Dubbin, Florence Bullough

**Affiliations:** 1 Department of Earth Sciences, The Natural History Museum, London, SW7 5BD UK; 2 Department of Earth Science and Engineering, Imperial College London, London, SW7 2AZ UK

**Keywords:** Siderophore, Al-goethite, Goethite, Enterobactin, Ferrichrome

## Abstract

Naturally occurring goethites often show Al for Fe substitution approaching 33 mol% Al. This substitution has potential to influence the rate of goethite dissolution and therefore the supply of bioavailable Fe. Siderophores such as ferrichrome and enterobactin have considerable potential to dissolve Fe from Fe^3+^ rich minerals, including Al-substituted goethites. Here, we show that Al substitution in synthetic goethites (0.021 ≥ *x* ≥ 0.098) gives rise to a significant increase in both ferrichrome- and enterobactin-mediated dissolution rates. In the presence of ferrichrome, Al-goethite (*x* = 0.033) yields a dissolution rate of 19.0 × 10^−3^ µmol m^−2^ h^−1^, nearly twice that of pure goethite, whereas dissolution of the most highly substituted Al-goethite (*x* = 0.098) is 36.9 × 10^−3^ µmol m^−2^ h^−1^, more than threefold greater than the pure mineral. Similarly, in the presence of enterobactin, the dissolution rate of Al-goethite increases with increasing Al substitution. Ferrichrome is a less effective ligand than enterobactin in its dissolution of both pure goethite and the range of Al-goethites, an observation we ascribe to the lower affinity of the hydroxamate functional groups of ferrichrome for both Fe^3+^ and Al^3+^. Despite greater affinity of both ferrichrome and enterobactin for Fe^3+^ over Al^3+^, we observe a broadly congruent dissolution of all our Al-goethites.

## Introduction

Soil goethites often show Al substitution for Fe, [Fe_1 − *x*_Al_*x*_OOH], where Al content can reach 33 mol% [i.e. Al/(Al + Fe) = 0.33] (Alvarez et al. 2007; Cornell and Schwertmann 2003; Schwertmann and Taylor 1989). This substitution changes unit-cell dimensions, crystal size, surface area, surface chemistry, domain morphology, and other structural properties that influence the mechanisms and rates of goethite dissolution (Ainsworth et al. 1989; Cornell and Schwertmann 2003; Schulze and Schwertmann 1984). Given the potential for Al substitution to influence the dissolution of goethite and other Fe(III) oxides, and thus the supply of bioavailable Fe(III), a growing body of work has explored the rates and mechanisms of dissolution for both natural and synthetic Al-substituted goethites (Cervini-Silva and Sposito 2002; Dominik et al. 2002; Ekstrom et al. 2010; Kukkadapu et al. 2001; Maurice et al. 2000).

The reductive dissolution of synthetic Al-goethites (where *x* = 0.051 or 0.33) by the Fe-reducing bacterium *Clostridium* *butyricum* revealed incongruent dissolution of Fe and Al and a rate of Fe release which decreased with increasing Al substitution (Bousserrhine et al. 1998). Accumulation of Al at the goethite surface during dissolution was thought to block the reactive surface sites, thus decreasing the rate of Fe release. In contrast, the reductive dissolution of a natural Al-goethite (*x* = 0.17) by *Shewanella putrefaciens* showed that dissolution was unaffected by Al sorption (Kukkadapu et al. 2001). In this latter study, reductive dissolution was thought to occur at sites distant from those of Al precipitation or adsorption. More recently, Ekstrom et al. (2010) have shown that the effect of Al substitution on reductive dissolution varies mainly with Fe(III) oxide type. For example, these workers observed no significant difference in Fe(III) reduction for either lepidocrocite or goethite over a range of Al substitutions. Rather, Fe(III) reduction rates were shown to vary only for ferrihydrite, with rates for pure ferrihydrite more than twice that for Al-ferrihydrite where *x* = 0.13. These conflicting studies demonstrate the considerable and ongoing uncertainty surrounding the effect of Al substitution on the reductive dissolution of Fe(III) oxides.

The dissolution of Al-substituted goethite has also been investigated for oxic environments. For example, when synthetic Al-goethite (0 ≤ *x* ≤ 0.088) was incubated under batch conditions (Maurice et al. 2000) with siderophore-producing bacteria (*Pseudomonas mendocina*), Fe release increased with increasing Al content, in contrast to the trends observed for reductive dissolution. Substitution of Al^3+^ for Fe^3+^ evidently increases the population of reactive surface sites which, under oxic conditions, enhances microbially mediated dissolution. Furthermore, a small number of studies have explored the release of Fe from Al-goethite in the presence of individual ligands such as oxalate and siderophores (Bousserrhine et al. 1999; Cervini-Silva and Sposito 2002). These latter ligands comprise a structurally diverse group of Fe(III) chelators secreted by microbes in response to Fe stress (Haselwandter 2008; Watteau and Berthelin 1994). The steady-state dissolution of synthetic Al-goethite (*x* < 0.10) in the presence of the trihydroxamate siderophore, desferrioxamine B (DFOB), has been examined by Cervini-Silva and Sposito (2002). These workers observed that the rate of DFOB-mediated Fe release increased with Al substitution and also with DFOB concentration. This latter effect was apparent only to 100 µM DFOB, above which a plateau in Fe release was observed.

Although DFOB is the siderophore most commonly used in batch dissolution experiments with Fe(III) oxides, including Al-substituted goethite, more than 500 other siderophore types have been identified, of which approximately half have been structurally characterised (Boukhalfa and Crumbliss 2002; Boukhalfa et al. 2003). Among the more notable of these are ferrichrome, a trihydroxamate fungal siderophore with a 1:1 stability constant, *K*_Fe(III_), of 10^29.07^ (Wawrousek and McArdle 1982) and enterobactin, a bacterial catecholate siderophore whose 1:1 stability constant with Fe(III) = 10^49^ (Loomis and Raymond 1991). Generally, both hydroxamate and catecholate functional groups show a lower affinity for Al^3+^ than for Fe^3+^. For example, acetohydroxamate forms a weaker 1:1 association with Al^3+^ (log *K* = 8.0) than with Fe^3+^ (log *K* = 11.4) (Martell et al. 2004), while catechol forms a weaker 1:1 complex with Al^3+^ (log *K* = 16.22; Sikora and McBride 1989) than with Fe^3+^ (log *K* = 20.0; Martell et al. 2004). Importantly, major divalent cations such as Ca^2+^ complex only weakly with ferrichrome (log *K* = 4.3) and enterobactin (log *K* = 4.6) (Hider et al. 1982).

Given the wide occurrence of ferrichrome and enterobactin in soils and other Earth surface environments, and their considerable affinity for Fe(III) as indicated by the high 1:1 stability constants, these two siderophores have potential to influence the release of Fe from Al-substituted goethites. Catecholate siderophores such as enterobactin are of particular interest due to their exceedingly high affinity for Fe(III) and the limited studies examining their interaction with Fe(III) oxides. The objective of our experiments was therefore twofold: to measure, for the first time, the dissolution kinetics of synthetic Al-goethite at pH 6.5 (1) as a function of Al substitution (*x* < 0.1) and (2) in the presence of either ferrichrome or enterobactin under steady-state conditions.

## Methodology

Pure goethite (α-FeOOH) and a series of four Al-substituted goethites were prepared as described in Schwertmann and Cornell (1991). Briefly, for the Al-goethite, we made fresh solutions of 0.5 M Al(NO_3_)_3_, 1 M Fe(NO_3_)_3_, and 5 M KOH. An aluminate solution was then prepared by combining 300 mL of KOH and 500 mL of Al(NO_3_)_3_ solutions while stirring constantly. Aliquots of the aluminate solution (20, 40, 80, and 120 mL) were then added to 2 L high density polyethylene (HDPE) bottles followed by addition of, respectively, 178, 176, 170, and 165 mL KOH stock solution. Immediately following addition of the KOH, 100 mL of 1 M Fe(NO_3_)_3_ was introduced to each bottle, which were subsequently brought to 2 L with deionised water. The suspensions were mixed for 10 min then incubated at 70 °C for 14 days and shaken daily. At the end of the 14 day reaction, the suspensions were centrifuged then washed three times with 1 M KOH to remove excess Al. The solids were washed with deionised water, freeze-dried, and stored in a dessicator at 23 °C for later use. As the goethites were dried and stored at temperatures well below the temperature at which dehydroxylation occurs (~110 °C), we avoid the formation of intracrystal micro- and mesopores observed by others (Naono et al. 1987; Ruan and Gilkes 1996).

**Fig. 1:**
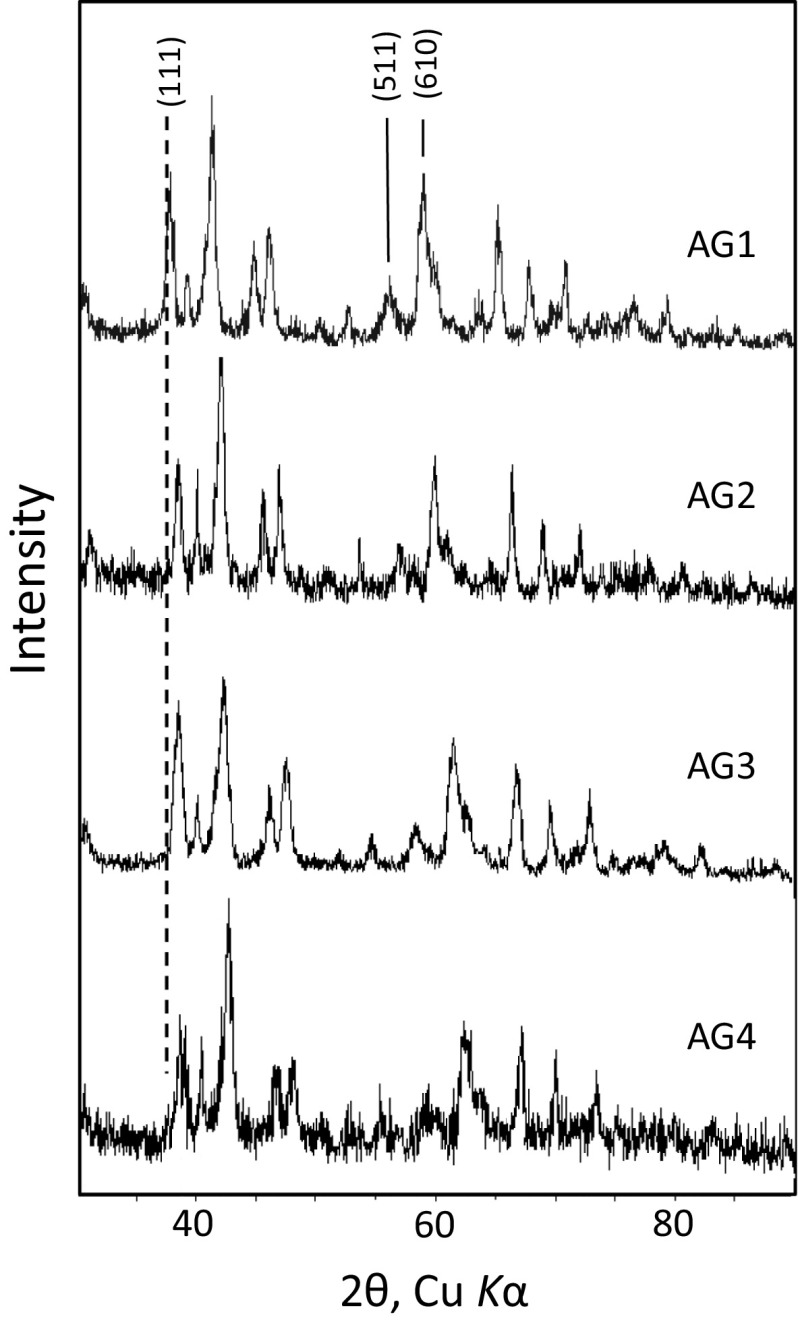
X-ray diffraction patterns for the four synthetic Al-goethites. Bragg reflections for the Al-goethites are indicated by the *hkl* indices, with progressive shifting of the 111 reflection to higher angles indicating increasing Al for Fe substitution. Samples AG1 through AG4 represent Al-goethites with, respectively, 2.1, 3.3, 4.6, and 9.8 mol% Al

The solids were confirmed as goethite and Al-substituted goethite by X-ray diffraction with an Enraf–Nonius PSD 120 diffractometer using Cu Kα_1_ radiation (45 mV, 45 kV) and fitted with an INEL 120° position sensitive detector. All samples appeared monomineralic (i.e. only goethite) at the detection limit of the XRD (~5 %). Ruan and Gilkes (1995) and Schwertmann and Cornell (1991) report smaller unit-cell dimensions for goethite following Al for Fe isomorphous substitution, shifting several peak positions to higher angles. We therefore interpret the progressive shift of the 111, 511, and 610 reflections to higher angles within the series of Al-substituted goethites as confirmation of increasing Al for Fe substitution in our goethites (Fig. 1).

The Al and Fe content of the goethites were determined by first digesting 100 mg samples of each oxide in 20 mL of 12 M HCl (80 °C) for 30 min or until dissolution was complete. The solutions were then diluted 100-fold with 10 % HCl prior to analysis for Al and Fe by ICP-OES (Varian Vista Pro, ICP Expert version 4.1.0). Surface area of the goethites was determined by N_2_-BET analysis following degassing of each sample with N_2_ for 24 h at 100 °C (Micrometrics Gemini III 2375). To ensure accuracy of surface area measurements, we analysed a reference kaolinite (15.9 ± 0.8 m^2^ g^−1^) alongside the goethite samples.

Acid dissolution experiments were conducted on the series of goethites to determine their dissolution kinetics and also to assess their Al substitution (Schwertmann and Carlson 1994). Briefly, 250 mg of each oxide were suspended in 250 mL of 6 M HCl (25 °C) with continuous stirring. Five mL subsamples of the suspension were removed periodically then passed through 25 mm Millipore^®^ nitrocellulose membrane filters (pore size 0.025 µm) into clear polythene tubes prior to Al and Fe analysis by ICP-OES. The reaction was allowed to proceed for 45 h.

**Fig. 2:**
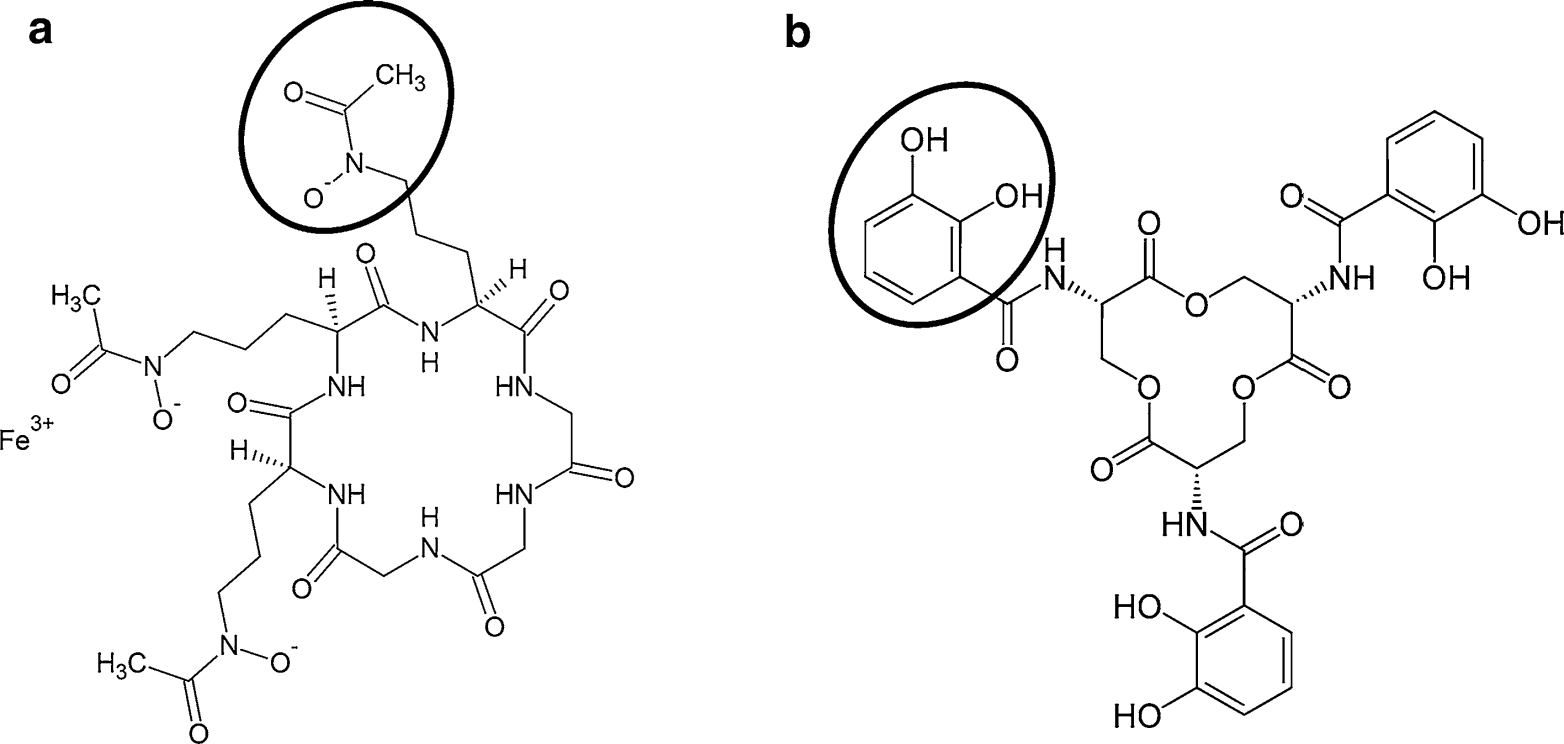
Structural representations of **a** ferrichrome and **b** enterobactin showing, respectively, their hydroxamate and catecholate functional groups. The proton dissociation constants for ferrichrome are: p*K*_a1_ = 8.11; p*K*_a2_ = 9.00; p*K*_a3_ = 9.83 (Anderegg et al. 1963), while those for enterobactin are: p*K*_a1_ = 6.0 ± 0.5; p*K*_a2_ = 7.5 ± 0.2; p*K*_a3_ = 8.6 ± 0.1 (Loomis and Raymond 1991)

The siderophore-promoted dissolution kinetics of pure goethite and the series of Al-goethites were measured in batch reactors at pH 6.5 and 25 °C. We chose pH 6.5 to ensure minimal proton-promoted dissolution during the 335 h reaction period and because it is representative of many Earth surface environments (e.g. soils) in which siderophores are abundant. The goethite concentration was 0.5 g L^−1^, and the background electrolyte was 10-mM NaNO_3_ (AnalaR, BDH) mixed with 1 mM MOPS [3-(*N*-morpholino) propanesulfonic acid, VWR] a noncomplexing pH buffer. We then added either iron-free ferrichrome [C_27_H_45_N_9_O_12_] or enterobactin [C_30_H_27_N_3_O_15_] (EMC Microcollection, Tübingen, Germany) (Fig. 2) to achieve an initial siderophore concentration of 240 µM, and pH was adjusted to 6.5 with 0.1 M NaOH or HClO_4_. We chose 240 µM as the initial siderophore concentration as it falls within a range of concentrations (20–1000 µM) commonly used in similar model systems, thus allowing comparison with results from other studies (e.g. Cervini-Silva and Sposito 2002; Cocozza et al. 2002; Wolff-Boenisch and Traina 2007; Stewart et al. 2013). Total volume of each batch reactor, prepared in triplicate, was brought to 200 mL by addition of background electrolyte. All samples were placed in a water bath at 25 °C for 335 h with constant stirring. Aliquots were periodically removed from each suspension then filtered through 25 mm Millipore^®^ membrane filters of pore size 0.025 µm. Supernatant solutions were acidified to pH 1.5 with HCl prior to analyses for Al and Fe by ICP-OES. Siderophore concentrations in the filtrate solutions were measured by the chelometric method as described in detail elsewhere (Cheah et al. 2003; Stewart et al. 2016). Briefly, concentrations of the Fe(III)–siderophore complex for each filtrate were measured spectrophotometrically by absorption at 467 nm and compared against those for a series of Fe(III)-siderophore standards, the latter containing predetermined quantities of enterobactin or ferrichrome to construct the calibration curve. Samples were then placed in 1-mL UV microcuvettes (10-mm path length) and absorbance at 467 nm measured on a Shimadzu UV-1800 spectrophotometer. The surface excess for each siderophore was then derived by dividing the enterobactin or ferrichrome lost from solution by the goethite surface area. On the basis of previous studies that show there is an optimal reaction period during which ligand adsorption is achieved, but where dissolution is minimal (Cocozza et al. 2002; Simanova et al. 2010), we choose 30 min reaction time to determine maximum siderophore surface excess.

The initial dissolution rates were derived by performing a linear least-square regression analysis. We chose the first five data points of the dissolution curve for the regression because they most closely approach linearity. The same number of points was used for each dissolution, giving regression coefficients (*R* ^2^) greater than 0.93 for all but one of the least-square fits.

## Results and Discussion

### Acid Dissolution of Synthetic Goethites

**Fig. 3:**
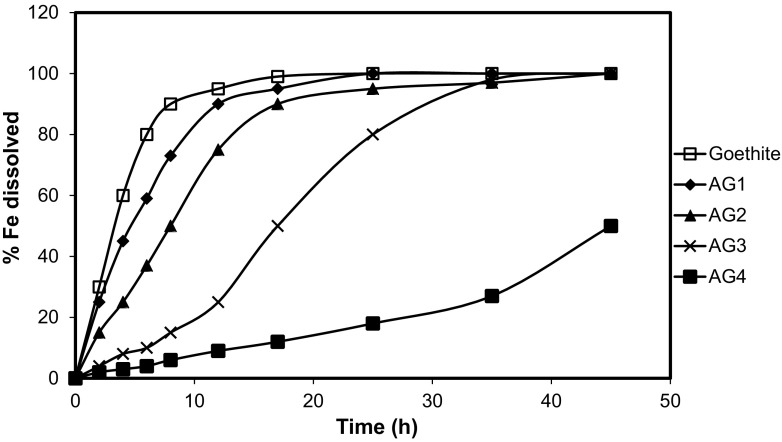
Dissolution-time curves of synthetic goethite and four synthetic Al-substituted goethites in 6 M HCl at 25 °C. Samples AG1 through AG4 represent Al-goethites with, respectively, 2.1, 3.3, 4.6, and 9.8 mol% Al

The Al content of the substituted goethites varied from 2.1 to 9.8 mol% Al (Table 1), while their surface areas decreased with increasing Al substitution, from 41.0 m^2^ g^−1^ for the least substituted Al-goethite to 29.7 m^2^ g^−1^ for the most highly substituted. Dissolution of these synthetic Al-substituted goethites alongside pure goethite in 6 M HCl yields S-shaped dissolution curves (Fig. 3). Generally, the shape of the curve became more sigmoidal, and the dissolution rate decreased with increasing Al substitution, a trend also observed by others (Cervini-Silva and Sposito 2002; Cornell and Schwertmann 2003; Schwertmann 1991). The shapes of our dissolution curves indicate that interdomain boundaries and surface defects may be created during the early stages of dissolution (Ruan and Gilkes, 1995), thereby increasing surface area and the rate of proton-promoted dissolution.

**Table 1:** Properties of the synthetic goethite and Al-goethite samples

Sample	mol% Al	Surface area (m^2^ g^−1^)	*t*_50_ (h)	
			Fe	Al
Goethite	0	43.2 ± 3.7	3.3 ± 0.5	3.8 ± 0.3
AG1	2.1 ± 0.4	41.0 ± 3.5	4.8 ± 0.4	4.5 ± 0.5
AG2	3.3 ± 0.9	35.6 ± 3.0	8.1 ± 0.7	9.3 ± 1.4
AG3	4.6 ± 0.8	33.1 ± 2.8	17.2 ± 1.4	15.9 ± 1.3
AG4	9.8 ± 1.5	29.7 ± 2.5	44.9 ± 2.2	44.2 ± 4.9

*t*_50_ = half-dissolution time for Fe and Al in 6 M HCl

Surface area determined by N_2_-BET

The time required to give 50 % dissolution of the solid, which we refer to as *t*_50_, is given for Fe and Al for each oxide and provides a measure of the rate of goethite dissolution (Table 1). Our data show that the rate of dissolution decreases with increasing Al substitution, an observation consistent with previous studies indicating that Al substitution in goethite reduces proton-promoted dissolution (Cervini-Silva and Sposito 2002; Cornell and Schwertmann 2003). Moreover, the observation that dissolution rate decreases also with decreasing surface area (Table 1) indicates that proton-promoted dissolution of our goethites is surface-controlled. Given the similarity of Fe_*t*50_ and Al_*t*50_ for each oxide, we infer that there is no significant difference in the rate of dissolution of these two cations from the solids. This observation also indicates that Al is broadly randomly distributed within the oxides.

### Siderophore-Promoted Dissolution

**Fig. 4:**
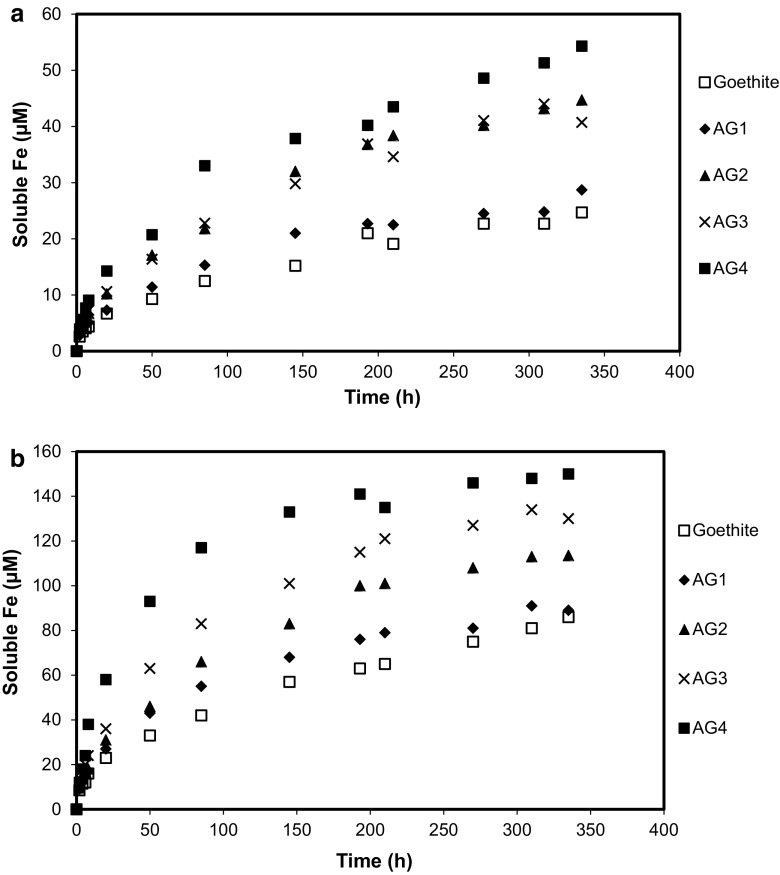
Iron release from synthetic goethite and four synthetic Al-substituted goethites in the presence of **a** ferrichrome and **b** enterobactin. Samples AG1 through AG4 represent Al-goethites with, respectively, 2.1, 3.3, 4.6, and 9.8 mol% Al. Initial siderophore concentration: 240 μM; solid concentration: 0.5 g L^−1^; pH 6.5; 25 °C

The Fe release kinetics for each oxide in the presence of ferrichrome or enterobactin at pH 6.5 and 240 µM initial siderophore concentration are shown in Figs. 4a, b, respectively. For both siderophores, the rate of Fe release is rapid initially then decreases at *t* > 20 h. Aluminium substitution in the oxides increases Fe release, with sample AG4 yielding the greatest soluble Fe at all reaction times for both ferrichrome and enterobactin. These observations are consistent with results from the dissolution of Al-goethite in the presence of DFOB (Cervini-Silva and Sposito 2002) and in the presence of siderophore-producing bacteria (Maurice et al. 2000). However, our results contrast with those for the dissolution of Al-goethite by Fe(III)-reducing bacteria (Bousserrhine et al. 1998; Ekstrom et al. 2010).

**Fig. 5:**
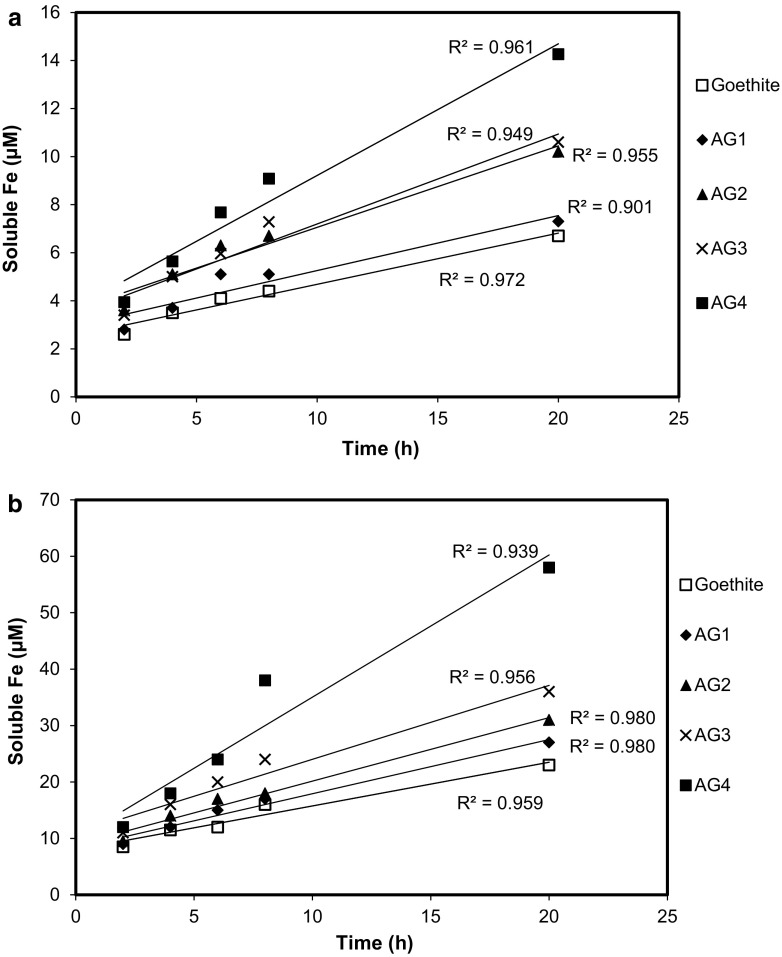
Initial iron release kinetics from synthetic goethite and four synthetic Al-substituted goethites in the presence of **a** ferrichrome and **b** enterobactin. Samples AG1 through AG4 represent Al-goethites with, respectively, 2.1, 3.3, 4.6, and 9.8 mol% Al. The lines through the data are linear least-square regressions (Table 2). Initial siderophore concentration: 240 μM; solid concentration: 0.5 g L^−1^; pH 6.5; 25 °C

The dissolution kinetics at *t* < 20 h (Figs. 5a, b) show that dissolved Fe concentration depends approximately linearly on time, which is typical when dissolution reactions are far from equilibrium (Lasaga 1998; Sposito 1994; Stumm and Furrer 1987). The slope of the regression line equation is therefore equal to the rate coefficient for Fe release (Table 2, column 3). The initial dissolution rates of pure goethite by ferrichrome and enterobactin are 9.91 × 10^−3^ and 35.9 × 10^−3^ µmol m^−2^ h^−1^, respectively. This compares with dissolution rates of 3.86 × 10^−3^ µmol m^−2^ h^−1^ (Cocozza et al. 2002) and 5.98 × 10^−3^ µmol m^−2^ h^−1^ (Stewart et al. 2013) for goethite in the presence of 240 µM DFOB. It is notable that enterobactin induces significantly greater initial dissolution rates for goethite than either ferrichrome (Table 2, column 4) or desferrioxamine B at equivalent siderophore concentrations.

**Table 2:** Linear regression equations, surface area normalised initial dissolution rates, surface excess for ferrichrome and enterobactin, and pseudo-first-order rate coefficients for dissolution of Fe(III) from goethite and Al-substituted goethite at pH 6.5 and 25 °C

Siderophore	Sample	Regression equation	Initial dissolution rate (µmol m^−2^ h^−1^)	Siderophore surface excess (µmol m^−2^)	Pseudo-first-order rate coefficient (h^−1^)
Ferrichrome	Goethite	*y* = 0.213*x* + 2.56	9.91 × 10^−3^	0.827	1.2 × 10^−2^
	AG1	*y* = 0.229*x* + 2.97	11.1 × 10^−3^	0.764	1.5 × 10^−2^
	AG2	*y* = 0.339*x* + 3.67	19.0 × 10^−3^	0.809	2.3 × 10^−2^
	AG3	*y* = 0.374*x* + 3.45	22.6 × 10^−3^	0.692	3.3 × 10^−2^
	AG4	*y* = 0.548*x* + 3.74	36.9 × 10^−3^	0.631	5.8 × 10^−2^
Enterobactin	Goethite	*y* = 0.775*x* + 8.00	35.9 × 10^−3^	0.871	4.1 × 10^−2^
	AG1	*y* = 0.960*x* + 8.32	46.8 × 10^−3^	0.616	7.6 × 10^−2^
	AG2	*y* = 1.13*x* + 8.90	63.2 × 10^−3^	0.558	11 × 10^−2^
	AG3	*y* = 1.31*x* + 10.9	79.2 × 10^−3^	0.680	12 × 10^−2^
	AG4	*y* = 2.52*x* + 9.84	170 × 10^−3^	0.713	24 × 10^−2^

Initial siderophore concentration = 240 µM

Solid concentration = 0.5 g L^−1^

*y* = soluble Fe (µM)

*x* = time (h)

Aluminium substitution gives rise to a significant increase in both the ferrichrome- and enterobactin-mediated initial dissolution rates. For example, in the presence of ferrichrome, sample AG1 (*x* = 0.021) yields a dissolution rate of 11.1 × 10^−3^ µmol m^−2^ h^−1^ whereas that for AG4 (*x* = 0.098) is more than threefold greater at 36.9 × 10^−3^ µmol m^−2^ h^−1^ (Table 2, column 4). Similarly, in the presence of enterobactin, the dissolution rate of the Al-goethites increases with Al substitution such that the rate for sample AG4 (170 × 10^−3^ µmol m^−2^ h^−1^) is approximately three times greater than that for sample AG1 (46.8 × 10^−3^ µmol m^−2^ h^−1^). Aluminium(III) for Fe^3+^ substitution evidently increases the population of reactive surface sites available for complexation by ferrichrome or enterobactin.

It is notable that there is little apparent difference in ferrichrome-mediated initial dissolution rates for goethite and sample AG1 (Fig. 5a), despite AG1 containing 2.1 mol% Al. However, when one considers the siderophore surface excess values for all samples, it is evident that the ferrichrome surface excess for goethite is about 10 % greater than that for AG1 (Table 2, column 5). When the dissolution rates are normalised for siderophore surface excess through a calculation of the pseudo-first-order rate coefficient, we observe an increase in dissolution kinetics in line with Al substitution. The pseudo-first-order rate coefficient is commonly used to characterise ligand-promoted dissolution kinetics far from equilibrium (Cocozza et al. 2002) and is obtained as a ratio by dividing the surface normalised initial dissolution rate by the surface excess of the ligand promoting the dissolution.

Steady-state Al release rates for each dissolution experiment are given in Table 3. If the Al-goethites dissolve congruently, the rate of Al release would vary with the Fe release rate according to the ratio *x*/(1 − *x*), where *x* is the Al mole fraction. This ratio for the Al-goethite samples AG1 through AG4 is 0.021, 0.034, 0.048, and 0.109, respectively. As shown in Table 3, the Al release rates derived experimentally for the Al-goethite samples in the presence of either ferrichrome or enterobactin approximate that predicted for the congruent dissolution of the oxide. Furthermore, the congruent dissolution we observe in the presence of these two siderophores corroborates our observation of congruence during proton-promoted dissolution in 6 M HCl (Table 1, columns 4, 5). These data together indicate that the Al in the Al-goethites is in solid solution with Fe and that there are no significant occurrences of gibbsite-like coatings or inclusions, even where *x* = 0.098 (i.e. for sample AG4), as these would give rise to differing responses to ligands and protons. Furthermore, given these data, we can infer that there is no significant secondary precipitation of Al or Fe released during dissolution of the solids.

**Table 3:** Steady-state Al release rates, and Al and Fe release rate ratios, for the Al-goethite samples in the presence of ferrichrome and enterobactin

Siderophore	Sample			
	AG1 (µmol m^−2^ h^−1^)	AG2 (µmol m^−2^ h^−1^)	AG3 (µmol m^−2^ h^−1^)	AG4 (µmol m^−2^ h^−1^)
Ferrichrome	0.289	0.532	1.15	4.69
Enterobactin	1.12	2.46	3.01	20.1
Release rate ratios				
$R_{\text{Fer}}^{\text{Al}}$/$R_{\text{Fer}}^{\text{Fe}}$	0.026	0.028	0.051	0.127
$R_{\text{Ent}}^{\text{Al}}$/$R_{\text{Ent}}^{\text{Fe}}$	0.024	0.039	0.038	0.118

*R* ^Al^ and *R* ^Fe^ in µmol m^−2^ h^−1^

*R* ^Fe^ values from Table 2

Initial siderophore concentration = 240 µM

Solid concentration = 0.5 g L^−1^

pH = 6.5

*Fer* Ferrichrome, *Ent* enterobactin

### The Efficacy of Enterobactin Versus Ferrichrome

Enterobactin is a more effective ligand than ferrichrome both in the magnitude of its effect (e.g. compare Fe release in Fig. 4a with that in Fig. 4b) as well as the consistency of its dissolution enhancement across diverse samples, from pure goethites to Al-goethites where *x* ~ 0.1 (i.e. sample AG4). The greater dissolution of goethite that we observe with enterobactin can not be ascribed to a greater initial ligand concentration, as both enterobactin and ferrichrome were present at starting concentrations of 240 µM. Likewise, the surface excess of the siderophores are broadly similar, varying from 0.558 to 0.871 µmol m^−2^ for enterobactin and 0.631 to 0.827 µmol m^−2^ for ferrichrome (Table 2). Differences in p*K*_a_ values for the two siderophores (Fig. 2) appear not to have significantly influenced adsorption. It is also notable that neither siderophore reaches surface saturation, with less than 10 % of the total initial ligand load sorbed to the oxide surface, similar to surface excess values reported by Wolff-Boenisch and Traina (2007) for enterobactin and DFOB. Furthermore, both siderophores are hexadentate, forming 1:1 octahedral complexes with Fe^3+^ where each organic molecule contributes three, bidentate functional groups that together satisfy the sixfold coordination of Fe^3+^.

In addition to each siderophore’s initial concentration, surface excess, and number of functional groups, another important property involved in the dissolution of oxides is the nature of the functional groups. Comparing data in Fig. 4a with that in Fig. 4b illustrates that the catechol functional groups of enterobactin are more effective than the hydroxamate groups of ferrichrome in dissolving both pure goethite and Al-goethite. The greater efficacy of enterobactin that we report here has been observed previously by others during the dissolution of metal oxides. For example, Brainard et al. (1992) observed during the dissolution of PuO_2_ that the rate constant in the presence of enterobactin was at least an order of magnitude greater than that for ferrichrome. Similarly, Wolff-Boenisch and Traina (2007) reported that enterobactin-promoted goethite dissolution rates at pH 6 are approximately five times greater than comparable DFOB-mediated dissolution rates. These results indicate that catechol containing siderophores are significantly more effective than their hydroxamate counterparts in the dissolution of various metal oxides.

Catechol siderophores, with p*K*_a1_ values of 6–8 for the dissociation of the first proton, are generally more acidic than hydroxamate siderophores, which have p*K*_a1_ values of 8–9 (Raymond et al. 1987). As protonation of the functional groups competes with metal chelation, the p*K*_a_ values of the donor groups must be considered when comparing the effectiveness of Fe(III) complexation by various ligands. Thus, given the lower p*K*_a1_ value for enterobactin (6.0 ± 0.5; Loomis and Raymond 1991) as compared to that for ferrichrome (8.11; Anderegg et al. 1963), one would predict greater affinity of enterobactin for Fe(III) at the pH of our systems (i.e. 6.5). Furthermore, as well as more favourable proton dissociation characteristics, enterobactin is uniquely predisposed to metal binding owing to the dynamic conformation of the free enterobactin molecule as described by Raymond et al. (2003). Specifically, the conformation of the uncomplexed ligand favours rapid initial binding of an Fe(III) atom, whereas the subsequent conformation change arising from proton loss and Fe(III) chelation promotes full encapsulation of the Fe(III) atom.

Thermodynamic parameters for Fe(III) complexes with enterobactin and ferrichrome are given in Table 4. For comparison, we also show data for the well studied trihydroxamate siderophore, DFOB. The significantly higher 1:1 stability constant for the enterobactin–Fe^3+^ complex (*K* = 10^49^), contrasts sharply with that for the two hydroxamate containing siderophores, namely the ferrichrome–Fe^3+^ complex (*K* = 10^29.07^) and the DFOB-Fe^3+^ complex (*K* = 10^30.60^). The *E*° values further demonstrate the greater thermodynamic influence conferred by enterobactin on the dissolution of Fe^3+^ from goethite, although it is apparent that all three siderophores induce considerable deviation from equilibrium.

**Table 4:** Siderophore–Fe^3+^ complex thermodynamic parameters

Ligand	log *K*	*E*° (mV vs NHE)	References
Enterobactin	49	−750	a, b
Ferrichrome	29.07	−448	c, d
Desferrioxamine B	30.60	−468	e, f

*K* = [FeL]/[Fe][L] for Fe + L = FeL (charges omitted for clarity)

^a^Loomis and Raymond (1991); ^b^ Harris et al. (1979); ^c^ Anderegg et al. (1963); ^d^ Wawrousek and McArdle (1982); ^e^ Cooper et al. (1978); ^f^ Spasojevic et al. (1999)

Given the very low, and broadly similar, surface excess values for both enterobactin and ferrichrome, it is plausible that the steep thermodynamic gradients created by these siderophores are rate controlling. The presence of powerful chelators such as enterobactin and ferrichrome creates a thermodynamic sink, with enterobactin in particular demonstrating a greater pull for iron release from goethite. Further, the apparent contradiction between the relatively high oxide dissolution rates combined with low surface excess values has been considered previously by Kraemer (2004) who concluded that the oxide dissolution reaction is influenced by both metal affinity phenomena as well as ligand adsorption factors. Our data are consistent with this view. However, given the magnitude of the stability constants and *E*° values, we attribute greater relevance to chemical affinity than to ligand adsorption.

Both the catecholate and hydroxamate functional groups show a lower affinity for Al^3+^ than for Fe^3+^. This is illustrated most clearly for catecholate by considering the model ligand, catechol, where the 1:1 Al^3+^-catechol complex gives a log *K* of 16.22 (Sikora and McBride 1989), and the 1:1 Fe^3+^-catechol complex has a log *K* of 20.0 (Martell et al. 2004). Likewise, the monohydroxamate analogue, acetohydroxamate, forms a weaker 1:1 association with Al^3+^ (log *K* = 8.0) than with Fe^3+^ (log *K* = 11.4) (Martell et al. 2004). These trends occur also for DFOB, the linear trihydroxamate siderophore, such that the log *K* for the 1:1 complex of Al^3+^ with deprotonated DFOB is significantly smaller than that of Fe^3+^ (Albrecht-Gary and Crumbliss 1998; Desroches et al. 1999):

$${\text{Al}}^{3 + } + {\text{DFOB}}^{3 - } = {\text{AlDFOB}}^{0} \quad \log K = 24.5$$

$${\text{Fe}}^{3 + } + {\text{DFOB}}^{3 - } = {\text{FeDFOB}}^{0} \quad \log K = 30.60$$

We attribute these trends in log *K* to Al^3+^ being a softer, less electronegative cation (*χ* = 1.61) than Fe^3+^ (*χ* = 1.96) and, therefore, less likely to form stable complexes with the hard-O donor atoms (O_D_) of either catcholate or hydroxamate. Also, because Al^3+^ has a lower electronegativity, this cation promotes less electron delocalisation along O_D_–Fe–O–Al linkages and, therefore, a lower bond energy for the neighbouring Fe–O groups. Despite these differences in Al^3+^ and Fe^3+^ affinity arising from the different functional groups of enterobactin and ferrichrome, we observe in this study a broadly congruent dissolution of Al-goethites at pH 6.5. Also, we note that both enterobactin and ferrichrome induce greater goethite dissolution as Al substitution increases over the range of Al contents examined in this study (i.e. 0.021 ≤ *x* ≤ 0.098). Clearly, further work is needed to elucidate the mechanisms underlying these various phenomena.
